# [Retracted] Interleukin-22 modulates cisplatin sensitivity of osteosarcoma cells by regulating the STAT3 signaling pathway

**DOI:** 10.3892/etm.2025.12964

**Published:** 2025-09-08

**Authors:** Zhiqiang Li, Renjie Xu, Xiangxin Zhang, Jun Shen, Guangxiang Chen, Tianming Zou, Xiao Yu

Exp Ther Med 19:1379–1387, 2020; DOI: 10.3892/etm.2019.8352

Following the publication of this paper, it was drawn to the Editor’s attention by a concerned reader that flow cytometric (FCM) assay data featured in Figs. 4B and 5B were strikingly similar to FCM data which were ultimately published in a number of other papers in different journals that were written by different authors at different research institutes, including an article that was received in advance of this one to the journal *Molecular Medicine Reports.*

Owing to the fact that the contentious data in the above article were found to be strikingly similar to data that have appeared elsewhere in other papers in the scientific literature, the Editor of *Experimental and Therapeutic Medicine* has decided that this paper should be retracted from the Journal. After contacting the authors, they accepted the decision to retract the paper. The Editor apologizes to the readership for any inconvenience caused.

